# Chronic Progressive Multiple Sclerosis – Pathogenesis of Neurodegeneration and Therapeutic Strategies

**DOI:** 10.2174/157015910792246218

**Published:** 2010-09

**Authors:** Dirk Fitzner, Mikael Simons

**Affiliations:** 1 Max-Planck-Institute for Experimental Medicine, Hermann-Rein-Str. 3, Göttingen, Germany; 2 Department of Neurology, Robert-Koch-Str. 40, University of Göttingen, Göttingen, Germany

**Keywords:** Multiple sclerosis, chronic progression, inflammation, neurodegeneration, treatment.

## Abstract

Multiple sclerosis (MS) is an inflammatory, autoimmune, demyelinating disease of the central nervous system (CNS) that usually starts as a relapsing-remitting disease. In most patients the disease evolves into a chronic progressive phase characterized by continuous accumulation of neurological deficits. While treatment of relapsing-remitting MS (RRMS) has improved dramatically over the last decade, the therapeutic options for chronic progressive MS, both primary and secondary, are still limited. In order to find new pharmacological targets for the treatment of chronic progressive MS, the mechanisms of the underlying neurodegenerative process that becomes apparent as the disease progresses need to be elucidated. New animal models with prominent and widespread progressive degenerative components of MS have to be established to study both inflammatory and non-inflammatory mechanisms of neurodegeneration. Here, we discuss disease mechanisms and treatment strategies for chronic progressive MS.

## INTRODUCTION

Multiple sclerosis (MS) is one of the most common neurological disorders frequently leading to permanent disability in young adults [1]. The clinical course is unpredictable and highly variable. In the majority of the patients a sudden onset of neurological deficits marks the beginning of the disease. In these patients the disease continues as relapsing-remitting MS (RRMS) characterised by episodes of acute exacerbations, followed by partial or complete recovery of the deficits. RRMS typically begins in the second or third decade of life and after a medium time to conversion of around 19 years approximately 70% of the patients subsequently develop secondary progressive MS (SPMS). The rate of conversion to SPMS is approximately 2-3% per year [2]. Secondary progression is usually defined as a period of clinical worsening, which is independent of relapses and sustained for at least six months. However, the time of conversion is sometimes difficult to pinpoint as it slowly builds up and remains unnoticed by the patient and the clinician for some time. Another challenge is to distinguish the chronic progression from residual symptoms that remain after patients have experienced acute relapses. There are no established biomarkers or definite clinical signs to discriminate between the relapsing-remitting and secondary progressive phase of MS.

Around 15% of the patients develop primary progressive MS (PPMS) characterized by the steady progressive deterioration in neurological function without preceding or concomitant relapses [3]. According to the revised McDonald diagnostic criteria from 2005 [4] the progressive neurological decline has to persist for at least one year and two of the following additional criteria have to be fulfilled: 1) nine MRI brain lesions or at least four brain lesions and abnormal visual evoked potentials, 2) at least two MRI spinal cord lesions, 3) CSF oligoclonal IgG bands or increased IgG index.

Characteristically, PPMS manifests around 10 years later than RRMS with a mean age of 39 years [3]. Interestingly, the median time of onset of SPMS and PPMS is almost identical [5]. In addition, the speed in which patients developed their neurological deficits in PPMS and SPMS is in remarkable accordance [5]. Once patients with SPMS and PPMS have reached Kurtzkes disability status score (DSS) 4 the progression onwards occurs in stereotype manner. In fact, a study of the natural history of MS of 1844 patients (Lyon Multiple Sclerosis Cohort) showed that while the beginning is highly variable, the progression of the disease is much more uniform [6]. The median time to reach DSS 6 was 23.1 years for patients with a relapsing-remitting onset and 7.1 for patients with primary progressive onset. However, the time to progress from DSS 4 to 6 or 7 was remarkably similar among the two subtypes. Once a clinical threshold of disability had been reached, neither the number of relapses nor the rate of progression was prognostic for the further clinical course. Additionally, relapses that occurred before reaching the threshold or the relapses that became manifest thereafter showed no significant influence on the progression of irreversible disability [6]. Thus, the onset of secondary chronic progression in MS does not seem to be solely determined by the inflammatory load that occurs and becomes apparent during the relapsing-remitting course of the disease.

These key studies of the natural course of MS raise a central question: How is chronic progressive MS triggered? In principal, three different scenarios are possible: First, inflammatory processes in the beginning of the disease determine the onset of chronic progression. Second, chronic progressive neurodegeneration starts from the beginning and inflammation occurs as a secondary response. A final possibility is that the inflammatory processes and chronic progressive neurodegeneration are not connected to each other and progress independently.

## BIOMARKERS OF CHRONIC PROGRESSIVE MS

While the question of how RRMS is connected to chronic progressive disease remains unanswered, there is accumulating evidence that both processes have distinct underlying pathogenic mechanisms. RRMS is regarded as an immune-mediated inflammatory demyelinating disorder, in which multifocal lesion are disseminated in time and space throughout the CNS [7, 8]. Lesions are associated with a breakdown of the blood-brain barrier and the presence of blood-derived lymphocytes and monocytes. Resolution of the inflammation, restoration of the conduction block and remyelination contribute to the clinical recovery. In contrast, chronic progressive MS is characterised by the irreversibility of the deficits due to progressive neurodegeneration [9-11].

To follow the different pathological processes of MS biomarkers and surrogate markers are required to monitor and predict the disease progression [12]. For RRMS magnetic resonance imaging (MRI) has proven to be a reliable tool to measure the formation of inflammatory lesions. Areas of focal inflammation are detected with high sensitivity as new gadolinium (Gd) enhancing lesions. Histological analysis at autopsy or after biopsy shows that Gd-enhancement indeed correlates with intense inflammatory activity and dense perivascular cuffs [13, 14]. In addition, Gd-enhancement lasts for 2-6 weeks similar to the duration of a typical relapse. The number of Gd-enhancing lesions and T2 lesions increases more rapidly in RRMS as compared to SPMS. Furthermore, patients with PPMS have fewer GD-enhancing lesions and a smaller T2 lesion load then patients with RRMS [15].

Thus, Gd-enhancement is a useful surrogate measure of relapses and therefore frequently used as an outcome criteria in many therapeutic trials of RRMS. Since six out of seven newly formed MRI lesions are clinically silent it is more sensitive as clinical outcome measures. However, the applicability of neuroimaging to predict long-term disability from the lesion load is not straightforward [16]. In fact, a longitudinal-study showed the EDSS score 14 years after diagnosis correlated only moderately with the lesion volume on MRI scans at 5 years (r=0.60) [17]. It seems that a change in the number and volume of the lesions correlates only with the concurrent change in disability at early but not at later stages of the disease.

Are there any surrogate markers of chronic progressive MS?

There are several MR measures available to determine neurodegeneration in MS. The two most specific MR methods are whole-brain atrophy measurements and MR spectrometry to measure the levels of the neuronal metabolite N-acetyl aspartate (NAA). Atrophy can be detected in both, the brain and the spinal cord of patients with SPMS and PPMS from the beginning of the disease and several studies have shown that atrophy correlates with disability [18, 19]. In addition to brain spinal cord volumetric measurements, MR spectrometry of NAA levels has demonstrated diffuse neuronal loss from the earliest clinical stages of disease [20-22]. Additionally, cerebral spinal fluid (CSF) biomarkers of neuronal damage such as neurofilament autoantibodies or the degree of neurofilament phosphorylation are beginning to emerge as new valuable surrogate markers for the neurodegeneration in MS [23, 24].

Furthermore, new genetic approaches such as genome-wide association scans for MS have recently identified new susceptibility loci for MS [25-27]. Such studies may also unravel genes associated with chronic progressive courses of the disease.

## NEURODEGENERATION IN PROGRESSIVE MS

Evidence for neuordegeneration in MS has been reported as early as 1868 by Charcot in post-mortem histolopathological analysis. Contemporary high-resolution laser-scanning confocal microscopy analyses have confirmed the presence of axonal pathology in MS lesions [28-30]. The most widely used marker for axonal dysfunction is the detection of amyloid precursor protein (APP) accumulations in the axons. Why APP becomes detectable in axons of MS lesions is not completely understood, but the breakdown of axonal transport followed by the subsequent retention of APP is the most likely possibility. It is also feasible that APP synthesis increases or degradation decreases as a result of the inflammatory process. Another immunohistochemical marker used to demonstrate axonal pathology is SMI32, an antibody that labels non-phosphorylated neurofilament in axons. The maturation of the axonal cytoskeleton is usually accompanied by increased neurofilament phosphorylation, a process that is induced by the myelinating glia and leads to an increase in axonal diameter [31]. Demyelination may trigger the dephosphorylation of neurofilaments in axons, thereby mediating structural changes within the axon. Other changes in the axonal cytoskeleton that are observed in MS lesions are the phosphorylation and aggregation of tau, a microtubule-binding protein [32, 33]. Increased carbonylation and degradation of cytoskeleton elements within axons have also been reported [34]. All these findings show that the axonal cytoskeleton is highly vulnerable and an important target in MS. These functional changes of the axonal cytoskeleton have important consequences if they persist as they will eventually lead to alterations in the transport of cargo along the axons and to an impairment of synaptic transmission [35].

Whereas these changes in the axonal cytoskeleton may mark a transient and still reversible dysfunctional state of the axon, other neuropathological findings such as axonal transsection and axonal end-bulb formation clearly show irreversible axonal damage in MS lesions [29]. Axonal swellings and transsections are already observed early in the disease within acute demyelinating lesions. More transsected or swollen axons were found within acute, inflammatory lesions as compared to chronic, sclerotic plaques [10, 36]. On the basis of this positive correlation between inflammation and structural changes in axons, the inflammatory process has been suggested to be responsible for the ongoing neurodegeneration in MS [37].

Besides this local, acute neuronal damage in inflammatory lesions, there is also a more widespread, diffuse neurodegeneration in the CNS of MS patients. MR spectrometry analyses of NAA levels reveal neuronal loss in normal appearing white (NAWM) and cortical grey matter [20, 38]. One possible explanation for the diffuse neuronal loss is secondary Wallerian degeneration as a result of focal axonal injury. However, there seems to be no correlation between total lesion load and the extent of neurogeneration as determined by NAA levels or brain atrophy measurement [12]. These finding raise the question whether the inflammatory attack by itself is the sole responsible factor or whether non-inflammatory mechanisms contribute to neurodegeneration in MS [10].

## MECHANISMS OF NEURODEGENERATION

There are a number of different inflammatory effectors that may be responsible for the axonal pathology in MS. For example, CD8^+^ T cells are able to exert cytotoxic effects on neurons within an inflammatory milieu [35]. Under physiological conditions neurons express low amounts of MHC class I. However, by inflammatory stimuli the expression of MHC class I is induced and this makes neurons highly susceptible to an attack by CD8^+^ T cells [39, 40]. In cell culture, the interaction of CD8^+^ T cells with axons resulted in transection of neurites and the formation of adjacent solitary neuritic spheroids similar as observed in active MS lesions [41]. Consistent with these findings, CD8^+^ T cells are frequently observed adjacent to damaged neurons in MS lesions [42]. The role of CD8^+^ T is further supported, by a study in a MHC class I light chain deficient mouse model of MS that shows extensive demyelination in the absence of an axonal pathology [43].

In addition to CD8^+^ T cells it is also likely that microglia participate in the neurodegenerative process in MS. The presence of activated microglia is a pathological hallmark of lesions in chronic progressive MS. These cells release a number of different factors that have been shown to be cytotoxic to neurons in culture [44]. For example nitrogen monoxide (NO) has repeatedly been seen to be detrimental to neurons for example by modifying ion channels, inhibiting mitochondrial respiration or blocking synaptic vesicle transport [45-47] . Neuropathological studies reveal an activation of the inducible form of NO synthase in acute lesions of patients with multiple sclerosis [48, 49].

Another important mediator of axonal damage seems to be glutamate as inflammatory stimuli may trigger glutamate release, which in turn induces excitotoxicity by calcium overload in neurons [50, 51]. Activation of AMPA and/or kainate receptors can damage axons or oligodendrocyetes [50, 52]. NMDA (N-methyl-D-aspartic acid) receptors are expressed on the surface of oligodendrocytes and in the myelin membrane and can, if activated abnormally, result in myelin degradation [53, 54].

Other inflammatory responses that can induce neuronal injury are antibody- and complement mediated processes. An interesting finding in this respect is the identification of autoantibody-mediated axonal injury by targeting neurofascin 186 (NF 186), a neuronal protein concentrated in the node of Ranvier [55]. Furthermore, contactin-2/TAG-1, a protein localized at the juxtaparanodal domain, has recently been identified as an autoantigen targeted by T-cells and autoantibodies in MS [56]. The contactin-2/TAG-1-directed autoimmunity induces encephalitis characterized by a preferential inflammation of the gray matter of the spinal cord and cortex [56].

A viral etiology of MS has been discussed [57, 58] and it feasible that specific viral infection may trigger an autoimmune-response towards axonal components and thereby contribute to neurodegeneration in MS.

Mitochondrial dysfunction is another factor that is likely to contribute to axonal damage in MS. In fact, the number of mitochondria is not only increased in chronic active and inactive lesions in progressive MS, but also the respiratory chain complex IV activity is altered [59, 60]. Oxidative damage to mitochondrial enzymes and DNA might be responsible for the impairment of mitochondria [61].

While it is clear that inflammation correlates with neuronal cell death in acute inflammatory lesions, permanent disability is low during the early stages of RRMS. In fact, neurological deficits seem to accumulate at a time when Gd-enhancing lesions become less frequent and the total lesion load remains stable.

One possible explanation for this discrepancy is that the MRI only maps the inflammatory lesions that are formed early in disease, but not the inflammatory infiltrates that occur later in the disease. There is indeed some evidence that the pattern of inflammation changes in the course of MS [62]. Lymph follicle-like structures in the meninges and in the perivascular space have been observed in the progressive phase of the disease [63]. In addition, while T- and B- cells are cleared from active lesions, there is a population of plasma cells that remain diffusely distributed within the brain parenchyma [62]. There is also an increasing number of microglia that are scattered throughout the brain in progressive MS [64]. It has been suggested that early inflammatory lesions trigger these changes [62] and create a new inflammatory environment with a different subset of inflammatory chemokines [65] which is formed within the CNS parenchyma. The inflammatory cells that persist in the CNS in progressive MS may directly induce neuronal damage. There is also evidence for an increasing number of cortical lesions in chronic progressive MS [11]. These lesions differ fundamentally from the white matter lesions as they are mainly composed of activated microglia and contain a much lower number of T- and B-cells [66-68]. It is possible that neuronal injury in the cortex is induced by a soluble factor released by the inflammatory infiltrates within the meninges.

A shift from adaptive to innate immunity with abnormally activated dendritic cells is another potential mechanism of disease progression in MS [9]. Maturation and activation of dendritic cells were found to drive a proinflammatory immune response in secondary progressive MS [69].

However, it is also possible that neuronal damage is indirect and a result of the ongoing demyelination in the brain. The loss of myelin has indeed far-reaching consequences for the axon. Demyelination does not only slow down nerve conduction, but also obliterates the axonal architecture and reduces long-term neuronal survival. The important function of myelin is highlighted in mouse mutants that are unable to form an intact myelin sheath as a result of gene deletions. For example, *Shiverer* mice contain a deletion of the MBP gene, which leads to the absence of myelin sheet formation and a severe behavioural phenotype with epileptic seizures and tremor [70]. The life span of these mice is dramatically reduced demonstrating the importance of myelin for the survival of an organism. *Shiverer* mice display a large number of changes in the axonal cytoskeleton and in the vesicular transport system that point towards a role of myelin in the regulation of fast axonal transport [71]. The breakdown of axonal transport that is often observed in neurons within active MS lesions could thus be explained in part by the loss of myelin ensheating the axons.

In addition to the role of myelin in structuring the axon, there is also evidence that myelin is essential for the long-term axonal survival [72, 73]. Evidence for such a function, comes again from mouse mutants that lack some of the major myelin genes. Knockout mice for the myelin-associated glycoprotein (MAG), the 2´,3´- cyclic nucleotide 3´- phoshodiesterase (CNP) and the proteolipid protein (PLP) form myelin and have an almost normal live span, but develop late-onset, chronic progressive neurodegeneration [74-76]. Axonal swellings, transections and an impairment of axonal transport occurning in these mice are highly reminiscent to the changes found in the CNS of patients suffering from MS. These mouse mutants provide evidence for a function of oligodendrocytes in providing trophic support for axons that is required for their maintenance into late adulthood. It will be important to identify these trophic factors and to determine whether they become limiting in chronic, progressive MS.

The concentration of voltage-gated sodium channels within specific regions between the internodes, the nodes of Ranvier, is another important function of myelin. The clustering of the sodium channels, Na_v_1.2 and 1.6, in the nodes of Ranvier is essential for the saltatory conduction of action potentials along the axons [77, 78]. The saltatory conduction does not only speed up nerve conduction several folds, but also conserves energy within the neuron. After a demyelinating event, the clustering of sodium channels in the nodes of Ranvier is lost and both, the Na_v_1.2 and 1.6 channels become diffusely distributed along the entire axon [79-82]. This response restores the conduction of the action potential, however resulting in a much higher energy demand. The increase in axonal sodium is compensated by enhancing the activity of the Na^+^/K^+^ ATPase thereby raising the requirement for ATP. Another consequence of high intra-axonal sodium levels is the reversal of the Na^+^/Ca^2+^ exchanger, which allows excess Ca^2+^ to enter the axon [83]. Ca^2+^ overload has severe consequences for the axon, as it results in cytoskeleton breakdown, activation of cell death pathways and increased proteolysis by the activation of Ca^2+^ sensitive proteases.

In summary, there is evidence for at least two different mechanisms that contribute to neurodegeneration in MS – axonal damage by a direct inflammatory attack and as a consequence of demyelination. These two different modes of actions may damage the axon at different stages of the disease. It is tempting to speculate that immune-mediated axonal injury occurs in active lesions and is responsible for an acute form of neurodegeneration, whereas demyelination induces late-onset neurodegeneration. In fact, available data indicate that axons do not to degenerate immediately after demyelination, but only when compensatory mechanisms fail and a threshold of damaging insults have occurred [72]. These different mechanisms of neurodegeneration have to be taken into account when designing neuroprotective treatment strategies for MS.

## THERAPY OF CHRONIC PROGRESSIVE MS

There has been tremendous progress in the treatment of RRMS over the past years. Several disease-modifying immunomodulatory or immunosuppressive drugs have already been approved and many more are currently in the last phases of clinical trials with promising outcomes [84]. Unfortunately, therapy of chronic progressive MS is lagging behind (summarized in Table **1**). So far, there is no proven or licensed disease-modifying drug to slow the progression of PPMS [85]. In fact, the outcome of most clinical trials evaluating the effect of immunomodulatory or immunosuppressive drugs in PPMS has been disappointing. By now the largest trial performed in patients with PPMS (943 patients) was carried out with the immunomodulatory drug, glatiramer acetate (GA) [86]. Whereas GA has been proven to be efficient in the treatment of RRMS, the trial with PPMS patients had to be terminated prematurely as an interim analysis showed no discernible effect in the disease progression after two years. Only a post hoc analysis suggested that GA may slow disease progression in a subset of patients. Trials with the immunomodulatory drug, interferon beta (IFN beta), in patients with PPMS have been of smaller size, but the outcome was similarly disappointing. Two small, single-centred, placebo controlled trials (123 patients) did not reveal any reduction of disability progression in PPMS patients [87, 88]. Although these studies were underpowered, to allow a definite conclusion on the efficacy of IFN beta the negative outcome did not encourage the initiation of larger trials. Recently, a single-center, phase two pilot study with interferon beta-1b on primary progressive showed no effect on sustained disability assessed by EDSS, but surprisingly revealed statistically significant differences for the Multiple Sclerosis Functional Composite score and for T1 and T2 lesion volume [89].

A small, phase II trial of the immunosuppressant agent, mitoxantrone was carried out in patients with PPMS using 12 mg/m^2^ of mitoxantrone or placebo every 3 months for 2 years. Again no benefit was detected of treatment on time to sustained disease progression [90].

There are also examples of effective treatment regiments for RRMS that have failed when these drugs have been tested in patients with SPMS. For example, the humanized monoclonal antibody, alemtuzumab (Campath-1H), which induces the cytolysis of CD52 positive cells leading to a T cell depletion and is highly effective in reducing relapse rate, MRI lesion load and disease progression in RRMS [91], does not seem to protect from disease progression once patients that have progressed to SPMS [92].

Another example is the immunosuppressant drug cladribine, which was not effective in modifying the disease progression in a placebo-controlled trial with 159 patients suffering from SPMS or PPMS, even though it produced and sustained significant reduction in the number and volume of Gd-enhanced lesions on MRI [93].

These and other trials indicate that there is a critical window of therapeutic opportunity in the treatment of MS with immunomodulating or immunosuppressive drugs [92].

A similar conclusion can be drawn when analyzing the efficacy of IFN-beta in trials conducted with SPMS. In the European trial of IFN-beta in SPMS, a reduction in relapse rates, MRI activity and progression of disability was observed [94, 95]. While reduced relapse rates were also observed in the North American and the SPECTRIMS trial of IFN-beta in SPMS, these trials failed to reproduce the beneficial effects on the progression of disability [94, 96]. The analysis of the study population revealed that the patients in the European trial were younger with a higher pre-study relapse rate, suggesting that the reason for the different outcomes lies in the more active inflammatory disease in the European trial. Furthermore, mitoxantrone has been shown to slow down the progression of disease progression in patients with active and rapidly progressive SPMS [97]. Again, predictive parameters of mitoxantrone effectiveness seem to be the number of relapses within the past year before treatment indicating that the inflammatory activity determines treatment response [98]. More recent trials with the B cell depleting monoclonal antibody rituximab showed an impact on disease activity in RRMS and neuromyelitis optica, but no efficacy in PPMS [99-101]. Even the aggressive treatment with autologous haematopoetic stem cell transplantation failed to suppress demyelination, neurodegeneration and clinical progression in the chronic progressive phase of the disease [102].

All of these studies indicate that the available approved therapies are most effective early in the disease when the pathophysiology is dominated by the inflammatory and not the degenerative component. The studies also suggest that most immunomodulatory and immunosuppressive therapies are unlikely to have any substantial effect once the disease has progressed into the chronic progressive phase of the disease. This raises the central question: if inflammation triggers neurodegeneration, why are current therapies not more efficient in slowing down disease progression?

One possible explanation is that current immunomodulatory and immunosuppressive are unable to target the inflammatory cells in progressive MS. This could be due to the compartmentalization of inflammatory cells behind the blood-brain barrier, which is not sufficiently permeable for most drugs [62]. It also feasible that changes in the inflammatory process have occurred which are not a target of the current immunomodulatory and immunosuppressive drugs [9]. In fact, most therapies are directed against the cells and mediators of the adaptive immune system, whereas cells of the innate immune system like activated microglia and dendritic cells are dominating in chronic progressive MS. Fingolimod, a sphingosine 1-phosphate receptor agonist, which has not only been shown to affect lymphocyte migration, but also to modulate dendritic cell maturation, might be a promising disease modifying drug for the chronic progressive phase of MS [103, 104].

Since the current approved disease-modifying therapies are most efficient early in the disease, it will be important to find out how effective they are in delaying the onset of chronic progressive MS. If there is indeed a causal relationship of inflammation and neurodegenerative, early immunomodulatory treatment should have indirect neuroprotective effects. In this regard, it is important to note in most patients disease-modifying therapy is usually initiated with IFN-beta or GA due to their favourable safety profiles, whereas the more effective drugs natalizumab and mitoxantrone are used as an escalating therapy. Although there is some hope that early initiation of IFN-beta [105] or GA delays the onset of chronic progressive MS, this has not been proven conclusively yet [106]. It might indeed be possible that more aggressive and early immunomodulatory or immunosuppressive treatments are required to substantial affect the time to progress to SPMS. Even though many approved disease-modifying anti-inflammatory drugs are available for the treatment of RRMS, new and more effective drugs with advantageous safety profiles will be required in the future.

It is also clear that drugs directly targeting neurodegeneration must be developed to efficiently treat chronic MS. There are already a relative large number of neuroprotective drugs that have been shown to be effective in different EAE models, but only few of them are currently being tested in clinical trials. The glutamate-antagonist riluzol is one promising candidate. A small pilot trial in 16 patients with PPMS showed neuroprotective effects on MRI parameters revealing a stablization of T1-hypointense lesion volume and cervical cord area [107]. Another example, is the Na^+^ and Ca^2+^ channel blocker, lamotrigine, which is currently been tested in trial including 120 patients with SPMS for 2 years. The compound MN-166 that inhibits leukotrien activity, phosphodiesterases and nitric oxide synthase seems to have neuroprotective effects as the analysis of an ongoing trial revealed significantly reduced loss of brain tissue volume and less new lesion evolution to persistent T1-hypointensties over 1 year. The tetracycline-derived compound minocycline was shown not only to mediate neuroprotective effects but also to modulate the activation state of microglial cells and to reduce leukocyte transmigration. Treatment with minocycline in the EAE model together with glatiramer acetate resulted in decreased disease activity and progression [108, 109]. The ability to prevent disease progression in PPMS and SPMS is also being evaluated for the cannabis extract, dronabinol.

Work in the EAE models have revealed additional drug targets for the treatment of neurodegeneration in MS. Blockade of AMPA-responsive glutamate receptors with AMPA antagonists or the treatment with a fullerene compound combined with a NMDA receptor antagonist ameliorate neurodegeneration in EAE [50, 52, 110]. Axonal protection has also been achieved using the Na^+^ channel inhibitors phenytoin and flecainide [111, 112]. Recently, the blocking of acid-sensing ion channel-1 with amiloride revealed a neuroprotective effect in EAE [113]. Another successful neuroprotective approach was the modulation of the b-nicotinamide adenine dinucleotide (NAD)-dependent pathway involved in Wallerian axonal degeneration in an EAE model [114]. Promising results have also been achieved by the treatment with recombinant erythropoietin in EAE and in a pilot trial with MS patients [115-117].

Another exciting approach is to promote remyelination in order to prevent axonal loss. The reasons why remyelination is impaired in MS are not complete understood, but one likely mechanism is the inhibition of oligodendrocyte precursor cells within chronic MS plaques [118, 119]. Proteome analysis of chronic plaques as performed by Han *et al.* [120] will be important to indentify the factors that block oligodendrocyte differentiation. One factor that has already been identified is LINGO-1 (Leucin rich repeat and Ig domain containing NOGO receptor interacting protein 1). LINGO-1 is a key inhibitor of oligodendrocytes differentiation [121] and LINGO-1 antagonism has recently been found to promote remyelination in animal models [122].

## CONCLUSION

Although tremendous progress has been made in the treatment of MS, there are still a number of important questions that need to be solved in order to develop more efficient drugs to treat chronic progressive MS in the future. It is still not known how chronic progressive MS is triggered. Although there is consensus that neuordegeneration is responsible for the chronic progression of disability, the mechanisms that induce neurodegenerartion are not known. For the development of future treatment strategies this question needs to be solved and new animal model that faithfully reproduce the neuropathological changes of MS must be developed. In most EAE models, acute, axonal damage within inflammatory lesions is observed, but the slowly evolving widespread neurodegeneration characteristic for chronic progressive MS is lacking. In addition, it will be important to define to what extent inflammation is responsible for the neurodegeneration in chronic progressive MS. Furthermore, research of non-inflammatory mechanisms of neurodegeneration will result in new discoveries that may open new avenues for the treatment of chronic progressive MS.

## Figures and Tables

**Table 1:** Treatment Trials in Progressive MS

Substance	Disease Course	Study Design	Status/outcome	Reference
rituximab	primary progressive MS	phase2/3 (efficacy, placebo controlled, randomized)n	no significant difference in time to confirmed disease progression, less increase in T2-lesion volume	[123]
natalizumab	primary progressive MS	phase1/2 (safety, efficacy, uncontrolled)	Recruiting	NIH clinical trial database
lamotrigine	secondary progressive MS	phase2 (efficacy, placebo controlled, randomized)	Completed	NIH clinical trial database
idebenone	primary progressive MS	phase2 (safety, efficacy, placebo controlled, randomized)	Recruiting	NIH clinical trial database
simvastatin	secondary progressive MS	phase2 (efficacy, placebo controlled, randomized)	Recruiting	NIH clinical trial database
dirucotide	secondary progressive MS	phase2/3 (efficacy, placebo controlled, randomized)	Completed	NIH clinical trial database
FTY720	primary progressive MS	phase3 (efficacy, placebo controlled, randomized)	active but not recruiting	NIH clinical trial database
interferon beta-1b	primary and secondary progressive MS	phase2/3 (efficacy, placebo controlled, randomized)	no effect on sustained disability assessed by EDSS, effect on MSFC score, reduced T2/1 lesions	[89]
cyclophosphamide	secondary progressive MS	phase3 (efficacy, vs methylprednisolone, randomized)	Recruiting	NIH clinical trial database
autologous mesenchymal stem cells	secondary progressive MS	phase1/2 (safety, efficacy, placebo controlled)	Completed	NIH clinical trial database
mitoxantrone	secondary progressive MS	phase3 (efficacy, dose controlled, randomized)	Completed	[97]
sunphenon EGCG	primary and secondary progressive MS	phase3 (efficacy, placebo controlled, randomized)	Recruiting	NIH clinical trial database
hematopoietic stem cell transplantation	secondary progressive MS	phase1/2 (safety, efficacy, uncontrolled)	ongoing but not recruiting	NIH clinical trial database
interferon beta-1a	primary progressive MS	phase2/3 (efficacy, placebo controlled, randomized)	no effect on sustained disability assessed by EDSS	[124]
ABT-874/human monoclonalantibody	secondary progressive MS	phase2 (efficacy, placebo controlled, randomized)	Completed	NIH clinical trial database
inosine	secondary progressive MS	phase2 (efficacy, placebo controlled, randomized)	Completed	NIH clinical trial database
cladribine	secondary progressive MS with active relapses	phase2 (safety, efficacy, placebo controlled, randomized)	ongoing but not recruiting	NIH clinical trial database
tovaxin autologous T cell vaccine	secondary progressive MS	phase1 (safety, efficacy, uncontrolled, not randomized)	trend for improvement, MRI lesions stable	[125]
interferon beta-1b and tacrolimus	secondary progressive MS	phase2 (safety, efficacy, dose controlled, randomized)	ongoing but not recruiting	NIH clinical trial database
teriflunomide (HMR1726)	secondary progressive MS	phase3 (safety, efficacy, dose controlled, randomized)	ongoing but not recruiting	NIH clinical trial database
erythropoetin	chronic progressive MS	pilot trial	Completed	[117]
immunoglobuline	primary and secondary progressive MS	phase3 (efficacy, placebo controlled, randomized)	no effect on disability assessed by EDSS	[126]
Fampridine	primary and seconary progressive MS	phase2 (safety, efficacy, dose controlled, randomized)	improvement in walking ability	[127]
treosulfan	secondary progressive MS with relapses	phase2 (efficacy, placebo controlled, randomized)	median number of relapses per year reduced, reduced number of Gd-enhancing lesions	[128]
